# Methadone-drugs interactions: possible causes of methadone-related deaths

**DOI:** 10.1186/2050-6511-13-S1-A2

**Published:** 2012-09-17

**Authors:** Vesna Mijatović, Isidora Samojlik, Stojan Petković, Nikša Ajduković

**Affiliations:** 1 Department of Pharmacology, Toxicology and Clinical Pharmacology, Faculty of Medicine, University of Novi Sad, 21000 Novi Sad, Serbia; 2 Department of Forensic Medicine, Faculty of Medicine, University of Novi Sad, 21000 Novi Sad, Serbia

## Background

Methadone is an effective analgesic and it is widely used to suppress withdrawal symptoms from other opiates. Its consumption is usually associated with concomitant drug use in heroin addicts, and this combination is a possible risk factor for lethal outcome. The aim of this study was to analyze characteristics of methadone-related deaths (MRDs), and to evaluate the concomitant use of the drugs that can contribute to methadone toxicity.

## Methods

To investigate MRDs, a 10-year retrospective study was carried out (2001–2010) at the Institute of Forensic Medicine in Novi Sad, Clinical Centre of Vojvodina, Serbia. These data included age and sex of subjects, and drugs detected in *post-mortem* samples of blood and urine. Toxicological screening and quantification of drugs were carried out in blood and urine using gas chromatography-mass spectrometry. Methadone concentration in blood was defined to be lower than 200 µg/L, 200–1000 µg/l, or higher than 1000 µg/L.

## Results

A total number of 40 MRDs was identified (19.1% of all deaths associated with fatal opiate-related poisoning). The median age of victims at the time of death was 31, whereas the majority of them (80%) were male. The concentration of methadone in blood and urine samples was quantified in 11 cases and in 9 of them it was lower than 200 µg/L (mean concentration 79.5 µg/L). In one case it was 245 µg/L, whereas in the other one methadone was detected only in urine in concentration of 1209 µg/L. In 7 cases only methadone was found (17.5 % of MRDs); 47.5% of MRDs was associated with other drugs – the average number of associated drugs was 3.5, while in blood samples of 35% of MRDs other illicit drugs were identified. The most frequent concomitants were one or more benzodiazepines (67.5% of MRDs), followed by antipsychotics (15%), tramadol (15%) and antidepressants (12.5%). The most commonly identified benzodiazepine was diazepam.

## Conclusions

In MRDs a low methadone level combined with other drugs was most frequently noted. The mechanism of death cannot be attributed to particular pathway. The most detected concomitants were well-known inhibitors, inducers or metabolic substrates of CYP3A4 and CYP2D6 involved in metabolism of methadone. Moreover, they can increase the risk of torsades de pointes and of respiratory depressant effects of methadone. Further studies could clarify the possible mechanism of death where methadone is used in combination with benzodiazepines in order to prevent MRDs.

